# Effect of material properties on emotion: a virtual reality study

**DOI:** 10.3389/fnhum.2023.1301891

**Published:** 2024-01-24

**Authors:** Cyril Bertheaux, Eliott Zimmermann, Mathis Gazel, Johanna Delanoy, Pierre Raimbaud, Guillaume Lavoué

**Affiliations:** ^1^Univ Lyon, Ecole Centrale de Lyon, CNRS, ENTPE, LTDS, UMR5513, ENISE, Saint-Étienne, France; ^2^Univ Lyon, Ecole Centrale de Lyon, CNRS, INSA Lyon, UCBL, LIRIS, UMR 5205, ENISE, Saint-Étienne, France; ^3^Univ Lyon, Centrale Lyon ENISE, Saint-Étienne, France; ^4^INSA Lyon, CNRS, UCBL, LIRIS, UMR 5205, Lyon, France

**Keywords:** emotion measurement, aesthetic emotion, smoothness, metalness, visual perception, virtual reality

## Abstract

**Introduction:**

Designers know that part of the appreciation of a product comes from the properties of its materials. These materials define the object’s appearance and produce emotional reactions that can influence the act of purchase. Although known and observed as important, the affective level of a material remains difficult to assess. While many studies have been conducted regarding material colors, here we focus on two material properties that drive how light is reflected by the object: its *metalness* and *smoothness*. In this context, this work aims to study the influence of these properties on the induced emotional response.

**Method:**

We conducted a perceptual user study in virtual reality, allowing participants to visualize and manipulate a neutral object – a mug. We generated 16 material effects by varying it metalness and smoothness characteristics. The emotional reactions produced by the 16 mugs were evaluated on a panel of 29 people using James Russel’s circumplex model, for an emotional measurement through two dimensions: arousal (from low to high) and valence (from negative to positive). This scale, used here through VR users’ declarative statements allowed us to order their emotional preferences between all the virtual mugs.

**Result:**

Statistical results show significant positive effects of both metalness and smoothness on arousal and valence. Using image processing features, we show that this positive effect is linked to the increasing strength (i.e., sharpness and contrast) of the specular reflections induced by these material properties.

**Discussion:**

The present work is the first to establish this strong relationship between specular reflections induced by material properties and aroused emotions.

## 1 Introduction

The selection of a material is an important step in the design of an industrial product. Many constraints must be taken into account when choosing a material (Ashby et al., 2007). These are mainly mechanical, physical, chemical, aesthetic, electrical, acoustic, thermal, optical, economic, environmental and perceptual characteristics (Karana et al., 2008, 2015; Ashby and Johnson, 2013). To choose the right material, engineers more easily focus their analyzes on the technical, functional, and economic aspects (Lindbeck and Wygant, 1995; Budinski, 1996); while designers are usually more attentive to the sensory and aesthetic properties of materials such as color, texture, sound, smell, taste (Zuo et al., 2001; Van Kesteren, 2008; Piselli et al., 2018). Thus, most of the characterization approaches of materials focused on functional and technical aspects, but recent studies showed that the attractiveness of a material depends on the semantic, expressive, sensory, emotional attributes of the object (Rognoli and Levi, 2004; Farhadi et al., 2009). These expressive and sensory qualities contribute to the overall perception of the product and are considered an important component of the purchasing decision.

Recent work attempted to explain how humans perceive the sensory properties of materials; which sensory modality dominates the product experience in the different stages of product use (Fenko et al., 2010); how the choice of a material contributes to the creation of “product value” (Doordan, 2003; Karana et al., 2010). But a large part of the interest in a material comes from its texture. For this reason, part of existing perceptual studies focused on the material experience by researching which component of the texture would be more likely influence the user experience. In this regard, Picard et al., 2003, proposed a multi-criteria scale for evaluating a texture by touch on five dimensions: texture, temperature, hardness, weight qualities and hedonic descriptor. Other authors focused their study on the relationships between tactile perceptions and surface properties of materials, highlighting some relationships between surface properties and sensory responses (Holliins et al., 1993; Chen et al., 2009; Okamoto et al., 2012). Some researchers used sensory metrology techniques to classify materials according to their viso-tactile properties or preferences (Olivo et al., 2013; Etzi et al., 2014; Sakamoto and Watanabe, 2017), therefore emphasizing the ability of humans to discriminate texture based on visual and/or tactile processing (Fenko et al., 2010).

At the same time, many authors also agreed that visuals do not provide the same information as touch (Violentyev et al., 2005; Bolognini et al., 2011; Xue et al., 2016). The influence of vision on other sensory modalities has also been very often demonstrated (Rock and Victor, 1964; McGurk and MacDonald, 1976; Pavani et al., 2000) and more particularly when it comes to evaluating the appearance of a material (Power and Graham, 1976; Lederman et al., 1986). Vision is the dominant modality when buying a product, because it is on the basis of visual information that the consumer compares and evaluates the products present on the market (Bertheaux et al., 2019). This particular sense allows us to make perceptual decisions by evaluating the aesthetics, quality, attractiveness of an object. Aesthetic judgments are then based on the observation of visual modalities that contribute to the representation of objects and their material characteristics (Rottenstreich and Shu, 2004). Thus, the “intuitions, feelings” an individual has toward the material properties of an object are important aspects that must be taken into account when designing an object. Nonetheless, it should be highlighted that materials can cause many kinds of emotions, and that it is possible to classify materials on the basis of their emotional value. Materials can therefore be classified according to 3 conditions: materials considered unpleasant such as sandpaper, materials considered neutral such as plastic, materials considered pleasant such as fur (Bertheaux et al., 2020). Images or material representation can also affect people emotionally.

Some researchers worked on image classification on cognitive and affective levels. To conduct studies on emotion, Lang et al. (1997) created an image library called IAPS (International Affective Image System). These images are of great interest because they have been classified into three categories: unpleasant, neutral, and pleasant. This library is accompanied by a list of average ratings of the emotions aroused by each image. This assessment was done to allow other researchers to select images that evoke a specific type of emotion. The evaluation of the IAPS images was carried out using the Self-Assessment Manikin tool, which assesses emotion on three dimensions: Pleasure (Valence), Intensity (excitement) and Dominance (Bradley and Lang, 1994). Participants were asked to rate whether they found this image pleasant, neutral, or unpleasant. This work highlights the power of the visual and the ability of visual material to arouse emotion. Other research consisted of evaluating the perceived visual quality of 3D graphical objects displayed on a screen or in virtual reality, but without questionning the aroused emotions (Nehmé et al., 2020; Lavoué et al., 2021). Other researchers were interested in the aesthetic emotions that we experience when we are seized by the beauty of a work of art, a form or a word (Nikolova et al., 2016). Finally Naz et al. (2017) show that the emotions produced by a *real* living space (affected by light, color, and texture) can be reproduced in a *virtual* environment. Other studies focused on material perception, by studying how the human brain estimates and analyzes physical parameters of materials (Fleming, 2014). Fleming’s results can already be used to identify the components that must be mastered in order to design products with an emotional appeal to arouse strong emotions in consumers. But very rare are the studies that propose to evaluate the effect of material visual properties on the induced aesthetic emotions.

In this context, the objective of the present study is to evaluate the effect of two visual material properties, metalness and smoothness, on the induced emotion produced when viewing and manipulating objects. Our study is the first to investigate the influence of these material properties on emotions. Our hypothesis is that there is a strong link between the perceptual properties of the object and hedonic judgment. For this, we built a virtual reality experience intended to measure emotional responses when manipulating an object with different material characteristics. The use of virtual reality allows us to isolate the effect of these visual characteristics from any other cofounding factors (e.g., the tactile sensation or the weight of the object). To explain the hypothetical effects of these characteristics on the aroused emotions, we also compute statistical image features, allowing to characterize the nature of specular reflections produced by these different material configurations.

The rest of the manuscript is organized as follows: first, the following section presents the theoretical background on visual and emotional processes involved in visual perception, and the related work. Then, section 3 details the materials and methods, while section 4 presents the obtained results. The statistical image features used to characterize the induced specular reflections are presented in section 5. Finally, we provide a brief presentation on the visual properties of materials capable of generating aesthetic emotions; after that, we explain why we selected the two optical properties (*metalness and smoothness*) to build our experiment. In a dedicated section, we describe the virtual environment and the protocol that we have implemented to study the emotional impact of a product during a manipulation phase of a 3D object. Finally, we end the paper with a discussion section as well as a brief conclusion.

## 2 Theoretical background and related work

Visual perception plays an important role when it comes to evaluating the beauty of a product. Several factors such as the material properties of the object influence feelings and make the product more attractive. In the following two subsections, we recall the visual mechanisms involved in the evaluation of the formal, textural, sensory, and aesthetic qualities of an object by highlighting the impact of the material properties in visual appreciation.

### 2.1 Visual perception and emotional reactions

According to Wolfson and Landy (1995), the visual perception plays a role in the analysis of visual content by distinguishing figure from background, estimating surface orientation, defining the shape, the color, the optical properties of objects and also to the perception of other visual stimuli. These visual processings begin in the eye, where the retina transforms light rays into electrical impulses that pass through the optic chiasmas, the optic pathways before reaching the subcortical visual nuclei, the primary visual cortex and the higher areas where subdivisions which separately and independently analyze the scene, the color, the form, the depth, the movement, the orientation before being reconstituted on the form of a coherent image (Livingstone and Hubel, 1988; Felleman and Van Essen, 1991; Moutoussis and Zeki, 1997). Part of the visual content travels via the occipito-temporal nervous pathway which connects the lateral geniculate body of the thalamus, to the primary visual cortex, to extrastriate areas, to the inferotemporal cortex, to the prefrontal area. The other part of the visual content takes the occipito-parietal nervous pathway which connects the lateral geniculate body of the thalamus, to the primary visual cortex, to the higher areas involved in visual processing. These two processing pathways participate in the reading of form, in the appreciation of the material properties of objects by analyzing different parts of the objects, in a given environment.

In humans, the visual system is the preferred way to decode the environment, to orient oneself, to detect dangers, to identify edible foods, but also to appreciate the aesthetics of a product, admire the beauty of a landscape or a person (Barrett and Bar, 2009; Tonder and Spehar, 2013). Visual functions are also known to create the aesthetic experience which contributes significantly to the creation of the hedonic judgments involved in the appreciation of aesthetic and perceived properties of objects (Lajoie and Delorme, 2003; Mottier, 2020). In this framework, part of the material experience depends on the texture as was described by Klatzky et al. (1989), but also by the unconscious response produced during the visual examination of textures (Landy and Bergen, 1991). Textural aspect of a surface can vary according to the intensity, the type, the position of the light sources, the optical properties of the material which constitutes the object as well as many other factors which can modify the rendering of the material. This change in appearance will strongly influence the judgment of the observer who will perhaps prefer one color more than another, one material to another, one form rather than another.

The product appearance plays an important role in the customer’s perception of quality and to be appreciated, a product must have an irreproachable appearance. The material properties of objects influence feelings, certain sensory qualities can make one product more attractive than another or improve aesthetics. Moreover, the existence of aesthetic emotions is known today (Leder et al., 2004; Scherer and Coutinho, 2013; Perlovsky, 2014). According to Beermann et al. (2021), aesthetic emotions are aroused by different sensory impressions generated by music, the arts, literature, theater, cinema, nature scenes or when we ask a person about their feelings after having participated in a moving visual experience. Aesthetic emotions refer to emotions that describe an experience as pleasant, amusing, moving or sometimes boring, disgusting. Menninghaus et al. (2019) define aesthetic emotions as subjective and intuitive evaluations having as their object aesthetic appeal or vice. This same author identified the four major characteristics of aesthetic emotions: (i) they include an aesthetic evaluation or appreciation of the respective events or objects, (ii) they are predictive of a certain type of aesthetic appeal, (iii) they are associated with a subjective feeling of pleasure or displeasure, and (iv) they are believed to predict liking or disliking the event or object in question. In a recent study, Silvia and Nusbaum (2011) concluded that it is in the field of art that we most often encounter aesthetic emotions by declaring that they experience aesthetic chills.

Research on aesthetic experience is carried out according to three approaches: (i) experimental research of aesthetics which studies feelings, preference, pleasure and beauty (Berlyne and Boudewijns, 1971; Armstrong and Detweiler-Bedell, 2008); (ii) research based on emotion psychology which studies emotional states, such as pleasure, interest, anger and disgust (Silvia, 2009); (iii) research that focuses its studies on somewhat unusual emotional states such as feeling moved, losing track of time, having a strong desire to cry, experiencing admiration, i.e., the manifestation of strong emotions provoked by visual stimulation (McCrae, 2007). Our research falls within the framework of the first approach by measuring the emotional reaction produced when seeing an object from everyday life. Starting from the observation that the aesthetic experience produced when observing a product depends in part on the material, we dedicated our experience to the study of the aesthetic emotions produced when viewing mugs presenting different material properties in a virtual environment. Raising the question: *are the material properties of an object sufficient to influence emotional feelings?*

### 2.2 Impact of the material properties in visual appreciation

Human beings have a great ability to recognize materials. Materials can take many different appearances depending on lighting, point of view and shape. According to Fleming (2014), during material perception, the human brain does not actually estimate the physical parameters of materials but is more adept at constructing “statistical generative models” that capture natural degrees of variation in appearance between samples. Thus, when determining perceived brightness, the brain does not estimate BRDF (bidirectional reflectance distribution function) parameters, but rather uses a constellation of low- and mid-level image measurements to characterize the extent to which the surface exhibits specular reflections. These statistical appearance models are more expressive and easier to calculate than physical parameters.

The color appearance of an object can be greatly affected by its physical parameters because the perceived color depends on the reflections produced by the spectrum of light. Humans consider color as an attribute of the object, interpreting the radiation they receive from this object in relation to what surrounds them (Thompson, 2003). Furthermore, colors play a role in the appreciation of a product. This attribute is taken into account in the purchasing decision by creating emotions and playing on preferences. Many papers deal with the association of color and emotion because the emotions aroused by colors affect daily life (Valdez and Mehrabian, 1994). For example, product colors positively or negatively influence consumer attitudes (Kotler, 1973; Albert, 2007; Jonauskaite et al., 2019). Wilms and Oberfeld (2018) showed in a study that changing the hue, brightness and saturation of a color could have an impact on the emotional and physiological response. By measuring participants’ emotional response using the non-verbal Self-Assessment Model Scale (SAM) (Bradley and Lang, 1994), they revealed that saturated and bright colors generated more Arousal; that hue had a significant effect on Arousal or Valence scores were highest for saturated and bright colors. Furthermore, several interaction effects of the three-color dimensions were observed for both Arousal and Valence, thus showing that object color parameters affect emotional feelings. Additionally, color is an important cue for object recognition.

Human beings also have a real ability to visually distinguish materials and deduce their properties. Thus, at a glance, humans recognize leather, fur, velvet, wood, plastic, elastomer, metal, stone, etc. The ability to categorize and recognize materials has already been studied. A first study consisted in showing subjects photographs representing different materials and asking the panel if they can identify them and classify them in the right family of materials (Sharan et al., 2009). A second study presented subjects with photographs of materials from different categories and asked them to rate various subjective qualities, such as hardness, shine, and beauty (Fleming et al., 2013). In both cases, the participants in these tests were able to recognize the material and classify it in the correct category based on their judgment and the study of their material properties. These researchers realized that in addition to being able to categorize and recognize materials, humans are able to anticipate the properties of materials without even having to touch them. For example: A future buyer can easily determine a phone case is hard/soft, smooth/rough, brittle/solid. To better understand this human ability to discriminate material properties through sight, a study was conducted to test the ability of subjects to distinguish photographs of “real” and “fake” materials. The results of these experiments showed that even with presentation times of only very short (40 ms), the subjects were able to distinguish between types of materials by observing the properties of the materials (Sharan et al., 2008).

At the same time, other researchers have studied whether humans are also capable of estimating and reproducing the visual properties of materials. For example, an experiment was conducted on the study of the reflectance properties of a surface (shininess). In this study, two computer-simulated “square” surfaces were presented side by side to a panel who were asked to evaluate the apparent level of reflection of the two surfaces. These two surfaces appeared similar even though the authors had modified the spatial spectrum and reflectance of one of the materials. The results showed that the constant error (difference between simulated and matched values) was large except when the two surfaces had the same shape parameters or when they differed only in scale. This observation suggests that the visual judgments of the subjects were based on the similarity of the luminance histogram of the surface image (Nishida and Shinya, 1998). Subsequently, Fleming et al. (2003), showed that the appreciation of glossiness occurs through differences in lighting. According to several other authors, the appreciation of the glossiness would depend on the binocular disparity, information of movement, properties of the reflections, the luminosity, the position and orientation of the light sources which will modify the appearance of possible shadows (Blake and Bülthoff, 1990; Wendt et al., 2008; Doerschner et al., 2011; Chadwick and Kentridge, 2015). On the other hand, a minor change in the properties of the material can lead to huge differences in the perceptual impression of gloss (Fleming, 2012). According to Marlow et al. (2012), the brain is not able to estimate the physical properties of surfaces. Instead, the brain searches for several clues that allow it to appreciate the visual qualities of the object viewed. In an experiment, these authors created samples that all had the same surface reflectance, but exhibited different depth reliefs and were generated with different illumination patterns. They thought observers would find the samples to be just as bright. But contrary to what they expected, the differences in lighting and reflections greatly influenced the perception of glossiness.

This work has highlighted human sensitivity regarding the appearance of materials. Industrial designers know that beyond technical and functional aspects, the choice of material will play a very important role in preferences. According to Fleming (2014), material appearance plays a disproportionate role in assigning value to things so precious materials fetch high prices, largely because of their shiny appearance.

Several studies have been devoted to the perception of metallic and non-metallic properties of materials. These studies have generally been carried out with digital representations of 3D objects and materials which were rendered and displayed on a computer screen (Lavoué et al., 2021). In modern graphics rendering pipelines, the physical surface properties that define the matte or glossy nature of an object are generally determined by two main parameters: the smoothness/roughness of the surface and the degree of metallicity (metalness). These two parameters plus the color, allow to faithfully reproduce the realistic appearance of a material, respecting its physical characteristics. These two material properties are interesting because they make it possible to strongly modify the appearance of a material. This change in appearance determines to what extent a material is metallic or not. Metallic materials tend to have a reflective surface, which makes them glossier, while non-metallic materials generally absorb more light, giving them a matte appearance. Smoothness refers to the micro-geometry of the surface. To obtain a matte material, the smoothness value must be as low as possible and to obtain a glossy material, the smoothness must be higher. Smoother surfaces will keep specular reflections focused, and they will appear to look brighter with more intense specular reflections when viewed from the proper angle. Conversely, matte materials tend to scatter light rather than reflect it coherently, creating a non-reflective surface. Varying metalness and smoothness properties allows to generate different and attractive visual characteristics, with different degrees of glossiness. Using these two metalness/smoothness material configurations is sufficient to test individual preferences and determine whether changing these two properties influences emotional responses.

Some authors have already been interested in the possible impact produced by the metallic aspects of certain products by suggesting that the metallic component can be used to create products with pleasant characteristics. Because metallic materials, such as stainless steel, aluminum, brass, copper are considered too often have a sophisticated and attractive appearance due to their smooth finish, shine and ability to reflect light coherently (Kim, 2017; Rebollar et al., 2017; Hasselgren et al., 2021). But these works do not provide enough information on the level of metalness or smoothness that should be used to design a very emotional material experience. In order to increase the visual satisfaction of visitors to four public art museums in the Jeju region (South Korea), Kim (2017), carried out visual evaluations of the museum’s decorative elements (colors and shapes) in the aim of determining whether they were in harmony with the environment. Their results show that the definition of the shape and color of decorative elements are important factors which are taken into account in visual satisfaction but this study does not give details on the decorative factors which give rise to the greatest satisfaction. For their part, Rebollar et al. (2017), studied how the visual appearance of chips packaging material (visuals, material, text) influences the perception of quality of a food product. The result of their study shows that these three design factors have an effect on quality perception. Concerning the choice of packaging material, their study consisted of studying the material effect produced by a “paper” bag with a matte finish and a bag with a “shiny metallic” finish. Although a very slight preference for the paper bag was noted, their results show that in this case the appearance of the “paper” vs. “metallic” material is not statistically significant. Hasselgren et al. (2021) and other authors cited above who are interested in the visual quality of digital models seek to optimize the appearance of 3D objects by playing on different variables such as: geometric simplification, appearance and shape of mesh joints, animation effects, rendering systems conversion and shading models; The other studies mentioned above are interested in better understanding how the brain perceives the optical and formal properties of materials and the environment. Thus, there are studies on visual perception, the emotional evaluation of museum equipment, packaging, the emotional evaluation of photos, but no study seems to seek the material parameters which are likely to generate the most positive emotions in a product design context. Furthermore, the emotional effects of material characteristics such as metalness and smoothness have never been studied.

To find out if the properties of the material produce different aesthetic emotions, we have designed an experiment in which we study the emotional reactions produced at sight, and during the manipulation of mugs using a prototype system realized in virtual reality. We generated different material appearances (from matte to glossy) by varying the values of metalness or smoothness. The emotional reactions produced by the mugs were evaluated on a panel of 28 people using Russell (1980) circumplex model, 1980, which measures emotion on two dimensions: arousal and valence.

## 3 Materials and methods

In the following subsection, we introduce the experiment which consists of studying the impact of material properties on induced emotional responses. First of all, we present the virtual environment which was created to immerse participants in a kitchen and offer them an immersive experience allowing them to handle mugs in a natural way. We introduce the rendering mode carried out on our 16 stimulus mugs and our scene before explaining the test protocol and the emotional evaluation method of the material properties of the mugs. We also provide information relating to our twenty-nine participants.

### 3.1 Virtual environment and stimuli

Our objective is to evaluate the impact of the visual appearance induced by two material characteristics (*metalness* and *smoothness*) on the emotions aroused. To isolate the effect of these characteristics from any other cofounding factors (e.g., the tactile sensation or the weight of the object), we conducted our experiment in virtual reality (VR). We developed an as-realistic-as-possible VR environment (see Section “3.1.1 Virtual environment”) allowing participants to manipulate different versions of a same object presenting different material characteristics and rate their induced emotion.

#### 3.1.1 Virtual environment

**FIGURE 1:**
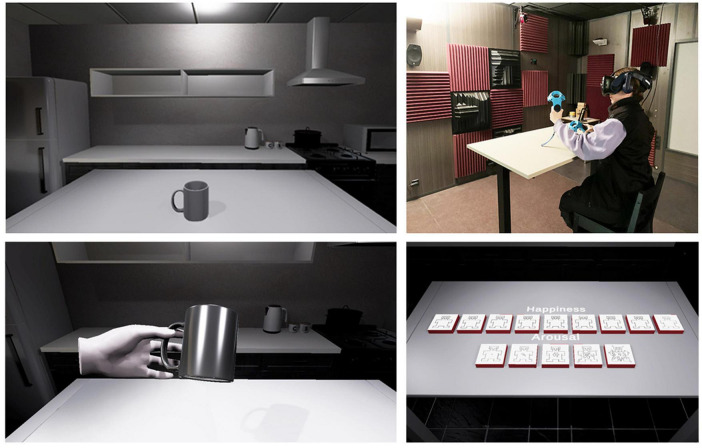
Virtual stage and physical setup.

We designed a virtual environment in which the use of mugs would seem plausible to VR participants. Therefore, we created a virtual 3D kitchen, where we placed the observer’s position on a raised bar chair facing a table, set at the height of a virtual kitchen island (Figure 1). For more realism, our virtual kitchen also included a refrigerator, cupboards, an oven, as well as containers such as cups or kitchen tools. The choice of these furniture, wall textures, floor coverings was made so that the kitchen would appear as familiar as possible to the participants, to immerse them as in real in their own kitchen with their own mugs.

Moreover, we chose to design this environment with shades of gray to keep the environment as neutral as possible (the environment is not the subject of this study). This choice is justified because it allowed us to focus our evaluation of emotions on the reactions produced in participants by the materials of the mugs, and not by the kitchen environment. This emotional effect could potentially have been due to the decorative elements selected to design our kitchen (Mahnke, 1996; Williams and Noyes, 2007).

Finally, to reinforce the feeling of presence and immersion, our virtual environment was designed to allow for a “pseudo-natural” interface since a real table and a real bar chair were placed at the same position and height than the virtual table and chair. Therefore, real haptic sensations could be felt by the participants when touching the table. To rate their emotion, they simply had to touch virtual buttons (see Section “3.2 Measures for users’ emotion”) placed on the table (see Figure 1).

#### 3.1.2 Stimuli creation

The choice to model a mug in 3D on the Solidworks 2022 software was determined by examining the IAPS (International Affective Image System) image library developed by Lang et al. (1997). As a reminder, this library includes 900 emotional images classified according to 3 emotional conditions. Among the images classified as *neutral*, we find photos of everyday objects such as a fan, a basket, a fork, a knife, a bowl, a spoon, and 2 mugs. One of the most neutral objects was a mug, and this particular object seemed interesting to us because it is a familiar object found in most kitchens. Moreover, the mugs are already made of different materials (plastic, ceramic, porcelain, metal, enamel), which is ideal for carrying out a study on the emotional impact produced by the properties of the materials. For more realism, we designed an interface allowing the participant to manipulate the mugs freely using an HTC VR controller. This manipulation, which took place in a kitchen environment, made it possible to examine the material properties of the object by placing it in different lighting, from different angles, observing the effect produced by the reflections.

The Unity 3D software with the HDRP (High-Definition Render Pipeline) rendering mode was used to render the scene, and the mugs. The choice of this rendering mode was motivated by the wish to maximize the visual quality and realism of the scene, the lighting atmosphere, and the visual aspect of the materials. The 3D mug model was used to create 16 mugs with different material effects obtained from the combination of 4 metalness values and 4 smoothness values.

**FIGURE 2:**
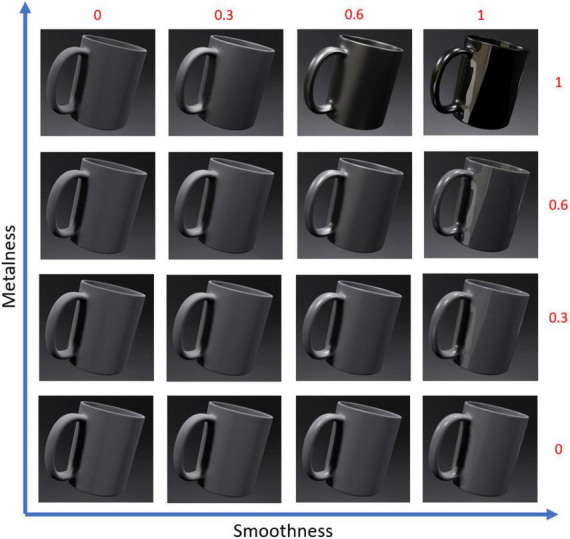
Realistic renderings of the 16 mug stimuli. On the abscissa, the level of smoothness from 0 (for a very rough material) to 1 (for very smooth material). On the ordinate, the level of metalness from 0 (for a very matte material) to 1 (for a very metallic material).

Finally, as our goal was to measure only the emotional response produced by metalness and smoothness, we removed all color information. This limited the risk of color influencing participants’ responses. The 16 mugs were modeled with the same gray level (level 128, out of 256 gray levels), consistent with the choice made for the virtual kitchen. Thus, the differences in appearance shown in Figure 2 are only due to changes in metalness and smoothness value (i.e., there were no black, gray or white cups, all were of 128 grays).

#### 3.1.3 Material configuration

The optical characteristics of a material are represented by the bidirectional reflectance distribution function (BRDF) which defines the spectral and spatial reflection characteristic of a surface. In real-time rendering engines, analytical models of BRDF are employed such as BlinnPhong (Blinn, 1977), GGX (Walter et al., 2007), or ABC (Löw et al., 2012). The Unity rendering engine that we use is based on the GGX BRDF model, and represents the basic material properties of a surface using 3 factors: metalness, smoothness, and base color (also known as albedo).

As stated in the Unity documentation, metalness and smoothness are defined as follows:

*Metalness:* when a surface is more metallic, it reflects the environment more and its albedo color becomes less visible. At full metallic level, the surface color is entirely driven by reflections from the environment. When a surface is less metallic, its albedo color is clearer and any surface reflections are visible on top of the surface color, rather than obscuring it.
*Smoothness:* Every light ray that hits a smooth surface bounces off at predictable and consistent angles. For a perfectly smooth surface that reflects light like a mirror, set this to a value of 1. Less smooth surfaces reflect light over a wider range of angles (because the light hits the bumps in the microsurface), so the reflections have less detail and spread across the surface in a more diffused pattern.”

### 3.2 Measures for users’ emotion

We use the Russel’s circumplex model to evaluate the emotional reactions produced by our 16 material effects. This model is based on the measurement of two dimensions: the *valence* and the *arousal* of the felt emotion. The valence corresponds to the self-evaluation of the emotional reaction produced by the visual stimulation. The arousal corresponds to the intensity, to the bodily awakening produced by the emotional reaction.

**FIGURE 3:**
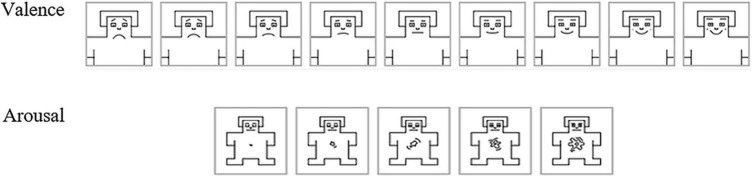
Self-assessment mankini – SAM, Bradley and Lang, 1994 (scale adapted to our test).

Such kinds of data are usually collected through paper questionnaire, however, to avoid here to break the immersion during virtual experiment, we designed an intuitive interface for our participants to self-assess and record their emotion directly in VR (see Figure 1, bottom-right). Participants could answer using the HTC controllers by touching virtual buttons (cubes) on the surface of the virtual table. A 9-point scale was used to the valence, and a 5-point scale for the arousal, represented by pictograms (see Figure 3). These scales were used referring to the SAM model – Self Assessment Mankini, which proposes to self-evaluate the emotion produced by a stimulation using two grids made up of small illustrations which represent valence and arousal (Bradley and Lang, 1994). The 9-point grid used to evaluate valence shows a happy face at one end and a sad face at the other (- 4 being unpleasant, 0 neutral, 4 pleasant). The 5-point grid used to evaluate the arousal shows at one end a simple point and at the other an explosion, which expresses the intensity of the emotional feeling (from 0-weak to 4-strong). Figure 3 shows the pictograms used for these two dimensions of emotion.

### 3.3 Participants

Twenty-nine participants (1 Queer, 7 women, 21 men) voluntary took part in our experiment, without compensation, with normal or corrected-to-normal vision, and aged from 16 to 77 (average 35, standard deviation 18). Each participant was informed about the procedure used for the experiment, signed a consent form, and was informed of their GDPR rights. In accordance with the applicable law relating to the “General Data Protection Regulation - GDPR,” they allowed for the use of their personal data in an anonymous way and only for the statistical purposes of this experiment. Finally, it should be highlighted that our participants were unaware that the main objective of this research was the evaluation of the effect of metalness and smoothness characteristics on their emotion.

### 3.4 Apparatus and experimental procedure

The hardware equipment consisted of a HTC VIVE PRO 2 virtual reality headset, and two HTC VR controllers to perform selection and manipulation actions in VR. The HTC Vive Pro 2 is a high quality headset with two high resolution screens (2448 × 2448 pixels per eye), a large field of view (up to 120 degrees) and a 120Hz refresh rate. Previous work (Toscani et al., 2019; Gil Rodríguez et al., 2022; Zaman et al., 2023) have validated the use of such kind of commercial headset for vision research.

The experiment took place in a dedicated room equipped with 4 HTC Vive base stations, to precisely track the motions of the VR headset and controllers and ensure high-quality user experience to our participants.

After having read the explanations regarding our experiment and completed the consent form, participants were asked to seat on a real raised bar chair, and to face a real table at the height of the VR kitchen table (see Figure 1). This realistic position and synchronization with the VR scene contribute to comfort and reinforce the feeling of immersion.

**FIGURE 4:**
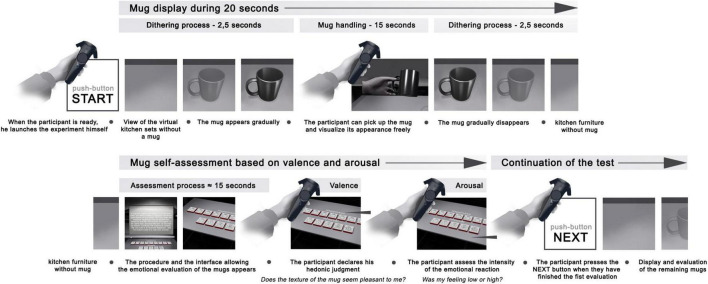
Experimental protocol.

Once the participant was correctly installed and feeling well and ready for the experiment, this one could start by pressing one of the HTC controller buttons (see Figure 4, top). First, a tutorial was presented to the participants, to allow them to try and repeat their two main actions for the experiment: (i) the handling and manipulation of the virtual mug, and (ii) the selection and validation of their declared emotion in terms of arousal and valence. Two training trials with two different mugs were presented to the participants; through this training phase, we ensured that all participants would perform the experiment with the same level of information and ease regarding the protocol and the use of virtual reality. Compared to the next trials, additional information was displayed in VR: virtual texts were providing explanations about the manipulation of the mugs, as well as information about the scales used to evaluate their emotion. Moreover, additional “Next” buttons were displayed, so that participants could have time to read all the text information given for the mug manipulation and emotion rating. Out of the training trials, no more “Next” buttons were necessary, and the next trial was automatically shown once the participant had rated their emotion. Nonetheless, the display and manipulation time of the mug was fixed to 20 s, with appearing and disappearing dithering animation effects (2.5 s additional time).

Regarding the emotional data collection (see Figure 4, bottom), two rows of virtual cubes were shown to the participants: the first one allowed them to express their hedonic judgment through the question “Does the material of this mug seem pleasant?” (Valence measurement, see Section “3.2 Measures for users’ emotion”), while the second one was used to assess the intensity of this feeling “Was my feeling low or high?” (Arousal measurement, see Section “3.2 Measures for users’ emotion”).

Then, participants had to perform the 16 experiment trials we designed, one for each mug created for this study. Our experiment followed a within-subject design, so all participants rated all our virtual stimuli. The presentation order of the mugs was randomized across all our participants to avoid any influence of this order on the results. Figure 4 summarizes our experimental protocol and the way it was shown to the participants.

## 4 Results

All statistical analyzes were conducted using R software. Extreme outlier values were excluded when values were below the 1st quartile - 3 × interquartile range (IQR) or above the 3rd quartile + 3 × IQR) – only one value in the valence data, and no values in the arousal one.

### 4.1 Classification of mugs by valence and arousal

**FIGURE 5:**
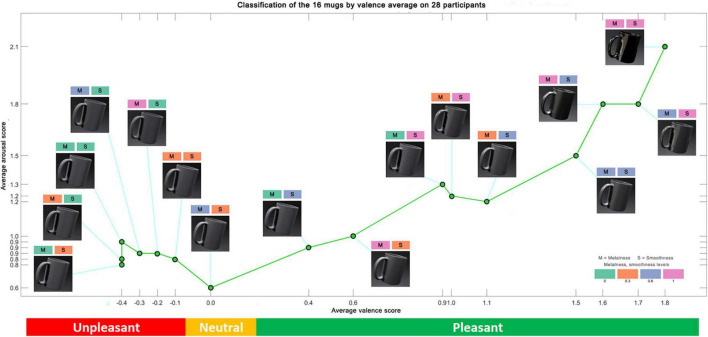
Classification of the 16 mugs by valence and arousal averaged on 28 participants.

The valence and arousal data collected during our experiment were averaged over the 28 participants for each mug. Figure 5 shows ranks of the mugs by valence from unpleasant to pleasant, with color codes representing their smoothness and metalness values. Mean valence scores are shown on the abscissa, mean arousal scores are shown on the ordinate.

We note that the valence scores obtained by the mugs considered unpleasant are very low, ranging from −0.4 to −0.0.1, i.e., values close to zero which could easily be assimilated to neutral values. But these mugs obtain slightly higher arousal scores (between 0.8 and 0.9) than the only mug judged to be neutral, which obtains a valence score of 0 and an arousal score of 0.6. Our hypothesis to explain these mugs that lead to negative responses, is that they seem rather unreal because they are too matte and too rough. These levels of metalness and smoothness can sometimes create an unpleasant impression that inspires a slight disgust. The levels chosen for the mug considered neutral do not lead to negative or positive responses that could be interpreted as emotional. The valence and arousal scores obtained by the mugs considered pleasant vary from 0.4 to 1.8 and from 0.4 to 2.1, respectively. These values are higher showing that certain materials lead to much stronger emotions of pleasure. Higher values are associated with mugs with higher smoothness and metalness. This impact of material characteristics is quantitatively assessed in the sections below.

### 4.2 Effect of the material on the average valence

The normality of the valence data was tested using the Shapiro-Wilk test. Considering that the distributions followed a non-normal law, and that the experiment was constructed based on a within-subject design in which the same group of participants was exposed to all levels of independent variables, we run a two-way repeated-measure ART (Aligned Rank Transform) ANOVA (Wobbrock et al., 2011; Elkin et al., 2021). At a risk of 5%, the test revealed that the smoothness factor has a significant effect on the measured valence (*F*(3,404) = 55.05, *p* = 2.22 e−16), same for the metalness factor (*F*(3,404) = 8.03, *p* = 3.2855 e−05). On the other hand, the interaction analysis shows that there is no interaction effect between the two variables (*F*(9,404) = 1.06, *p* = 0.38785). Which indicates that there is no relationship between the variable smoothness and the variable metalness.

**FIGURE 6:**
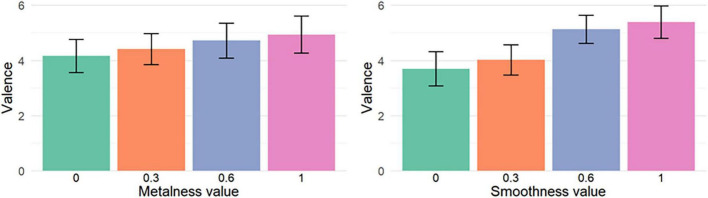
Mean valence for each value of metalness (left) and smoothness (right). The error bars show the 95% confidence intervals. Valence has been rescaled from [–4, 4] to [0, 8] for this particular figure.

**FIGURE 7:**
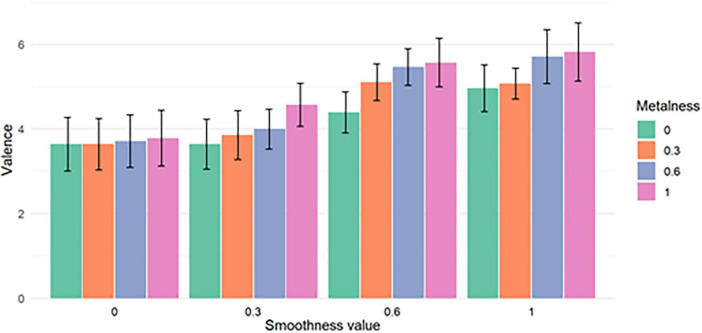
Mean valence for each combination of metalness and smoothness values. The error bars show the 95% confidence intervals. Valence has been rescaled from [–4, 4] to [0, 8] for this particular figure.

This influence of material characteristics on reported valence scores is illustrated on Figures 6, 7. Figure 6 shows the mean valence averaged for each value of metalness (left) and smoothness (right) (Figure 6), while Figure 7 shows the mean valence averaged for each metalness/smoothness combination (Figure 7).

We performed Tukey *post hoc* tests between each pair of smoothness and metalness levels. The Tukey correction was applied on the *p*-values obtained by the analysis.

First, we observed the valence ∼ metalness relationship (see Table 1). Significantly different pairs are: Metal 0 – Metal 0.6 (*p*-value = 0.0110, *t* = −3.102); Metal 0 – Metal 1 (*p*-value ≤ 0.0001, *t* = −4.628); Metal 0.3 – Metal 1 (*p*-value 0.0092, *t* = −3.161).

**TABLE 1:** Tukey *post hoc* tests for the valence dependent variable between metalness levels.

Pair compared	*t*-value	*p*-value
Metal 0 – Metal 0.3	-1.456	0.4652
Metal 0 – Metal 0.6	-3.102	0.0110
Metal 0 – Metal 1	-4.628	<0.0001
Metal 0.3– Metal 0.6	-1.639	0.3581
Metal 0.3 – Metal 1	-3.161	0.0092
Metal 0.6 – Metal 1	-1.526	0.4231

Next, we observed the valence ∼ smoothness relationship (see Table 2). Significantly different pairs are: Smooth 0 – Smooth 0.6 (*p*-value ≤ 0.001, *t* = −8.819); Smooth 0 – Smooth 1 (*p*-value ≤ 0.001, *t* = −10.524); Smooth 0.3 – Smooth 0.6 (*p*-value ≤ 0.001, *t* = −7.368); Smooth 0.3 – Smooth 1 (*p*-value ≤ 0.001, *t* = −9.077).

**TABLE 2:** Tukey *post hoc* tests for the valence dependent variable between smoothness levels.

Pair compared	*t*-value	*p*-value
Smooth 0 – Smooth 0.3	-1.451	0.4683
Smooth 0 – Smooth 0.6	-8.819	<0.001
Smooth 0 – Smooth 1	-10.524	<0.001
Smooth 0.3 – Smooth 0.6	-7.368	<0.001
Smooth 0.3 – Smooth 1	-9.077	< 0.001
Smooth 0.6 – Smooth 1	-1.727	0.3112

### 4.3 Effect of the material on the average arousal

The normality of the arousal data was tested using the Shapiro-Wilk test. Considering that the distribution follow a non-normal law and given that the experiment was constructed based on a within-subject design in which the same group of participants is exposed to all levels of independent variables, we run a two-way repeated-measure ART (aligned rank transform). At a risk of 5%, the test revealed that the smoothness factor has a significant effect on the measured Arousal (*F*(3,405) = 33.8959, *p* = 2.22 e−16), same for the metalness factor (*F*(3,405) = 11.9944, *p* = 1.5419 e−07). Moreover, the interaction analysis shows that there is an interaction effect between the two variables (*F*(9,405) = 3.3416, *p* = 0.00058595).

**FIGURE 8:**
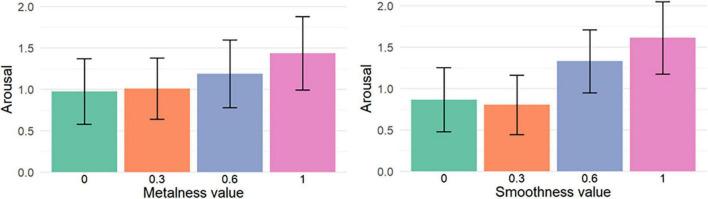
Mean Arousal for each value of metalness **(left)** and smoothness **(right)**. The error bars show the 95% confidence intervals.

**FIGURE 9:**
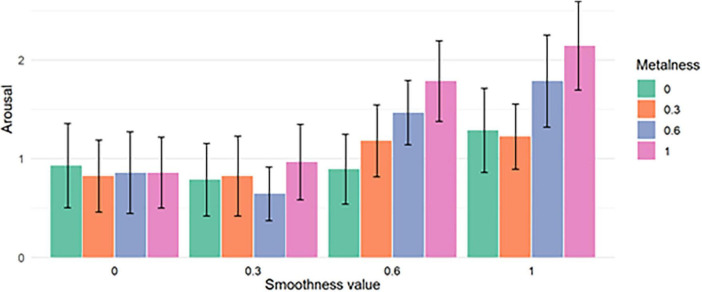
Mean Arousal for each combination of metalness and smoothness values. The error bars show the 95% confidence intervals.

This influence of material characteristics on reported arousal scores is illustrated through Figures 8, 9. Figure 8 shows the mean arousal averaged for each value of metalness (left) and smoothness (right), while Figure 9 shows the mean Valence averaged for each metalness/smoothness combination.

We performed Tukey *post hoc* tests between each pair of smoothness and metalness levels. The Tukey correction was applied on the *p*-values obtained by the analysis.

First, we observed the arousal ∼ metalness relationship (see Table 3). Significantly different pairs are: Metal 0 – Metal 0.6 (*p*-value = 0.0042, *t* = −3.394); Metal 0 – Metal 1 (*p*-value ≤ 0.001, *t* = −5.591); Metal 0.3 – Metal 1 (*p*-value = 0.0001, *t* = −4.278).

**TABLE 3:** Tukey *post hoc* tests for the arousal dependent variable between metalness levels.

Pair compared	*t*-value	*p*-value
Metal 0 – Metal 0.3	-1.313	0.5550
Metal 0 – Metal 0.6	-3.394	0.0042
Metal 0 – Metal 1	-5.591	<0.001
Metal 0.3 – Metal 0.6	-2.080	0.1612
Metal 0.3 – Metal 1	-4.278	0.0001
Metal 0.6 – Metal 1	-2.198	0.1256

Next, we observed the arousal ∼ smoothness relationship (see Table 4). Significantly different pairs are: Smooth 0 – Smooth 0.6 (*p*-value ≤ 0.001, *t* = −5.882); Smooth 0 – Smooth 1 (*p*-value ≤ 0.001, *t* = −7.756); Smooth 0.3 – Smooth 0.6 (*p*-value ≤ 0.001, *t* = −6.247); Smooth 0.3 – Smooth 1 *p*-value ≤ 0.001, *t* = −8.121).

**TABLE 4:** Tukey *post hoc* tests for the arousal dependent variable between smoothness levels.

Pair compared	*t*-value	*p*-value
Smooth 0 – Smooth 0.3	0.365	0.9834
Smooth 0 – Smooth 0.6	-5.882	<0.001
Smooth 0 – Smooth 1	-7.756	<0.001
Smooth 0.3 – Smooth 0.6	-6.247	<0.001
Smooth 0.3 – Smooth 1	-8.121	<0.001
Smooth 0.6 – Smooth 1	-1.874	0.2408

## 5 Objective characterization of specular reflections

**FIGURE 10:**
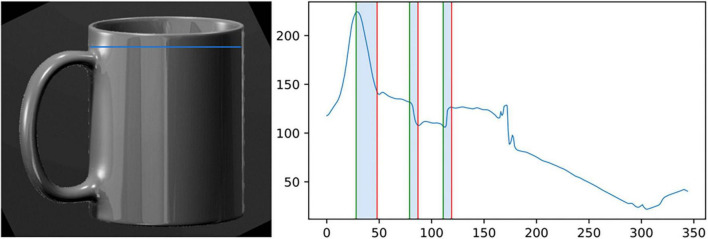
The rendering of a mug **(left)** with the line (in blue) that we use to compute the luminance profile shown in right. The three highlighted areas correspond to the transitions between high-highlight and no-highlight areas. These widths are used to compute the highlights sharpness feature.

To explain the positive effects of material characteristics on the aroused emotions, we hypothesize a strong link with the strength (i.e., the intensity) of specular reflections induced by the light-material interactions. To validate this hypothesis, we evaluate the strength of specular reflections using computer vision statistics computed on rendered images of the mugs. More precisely we follow the approach from Di Cicco et al. (2019) and Delanoy et al. (2021), illustrated in Figure 10: for each image we extract the luminance profile which runs through the most luminous and sharp highlights (the position of this line of interest is the same for all images), then we extract the two features evaluated as the most relevant by both authors to characterize the strength of reflections: *Michelson contrast* (Michelson, 1927) obtained as *(Imax-Imin)/(Imax* + *Imin)* and *highlights sharpness* which corresponds to the mean width of the highlights transitions (see Figure 10). Those two features are computed on the 16 mug images and normalized such that they both have a mean of 0 and a standard deviation of 1.

We then computed the “strength of reflections” predictor proposed by Delanoy et al. (2021) which is linear combination of *Michelson contrast* and *highlights sharpness* with weights resp. equal to 0.6 and 0.4.

**FIGURE 11:**
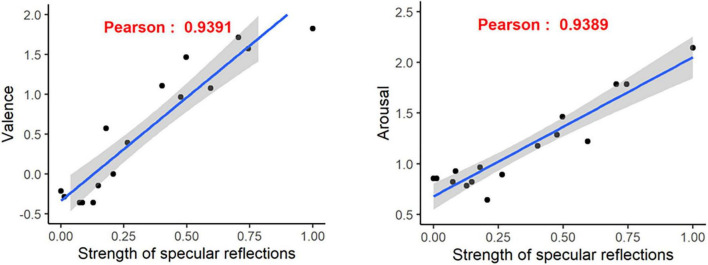
Scatter plots of mean valence **(left)** and arousal **(right)** value vs. specular reflection strengths. Corresponding linear regression models and Pearson correlation values are provided.

Figure 11 illustrates the relations between this predictor and the arousal and valence values reported for each mug. We obtain very strong relationships with significant Pearson correlation around 0.94 for both valence and arousal.

## 6 Discussion

The graph presented in Section “4.1 Classification of mugs by valence and arousal” ranks mugs by emotional value. In our study, we notice that 6 mugs are considered as *unpleasant*, one mug as *neutral*, and 9 mugs as *pleasant*. Most *unpleasant* mugs are obtained for lowest metalness and smoothness values, which produce very matte visual aspects. On the contrary, the most *pleasant* mugs (with both high arousal and valence values) are associated with highest metalness and smoothness values. Those positive effects of both metalness and roughness on valence and arousal are confirmed by the statistical analysis presented in Sections “4.2 Effect of the material on the average valence and 4.3 Effect of the material on the average arousal.”

The lack of attractiveness of mugs associated with low metalness/smoothness values could be explained since, as orally reported by the participants, these mugs seemed them bland and gave them the impression of an unnatural or degraded 3D object. Indeed, these “non-metallic and rough” mugs reflect very little light and have a very matte appearance that produce very few reflections. This absence of reflections creates a feeling of dissatisfaction because this material configuration does not provide enough visual cues to allow the user to fully distinguish the object’s shape, whose edges appear slightly blurred.

One mug was rated as *neutral* in appearance with an average valence score of 0 and the lowest arousal score of the series (0.6). This result indicates that the material configuration used to generate this mug was neither unpleasant nor pleasant, that is to say emotionally neutral.

The 9 mugs considered as pleasant own average valence scores between 0.4 and 1.8 and average arousal scores that can go up to 2.1. This indicates the “metallic and smooth” material configurations evoke the most positive emotions. Our hypothesis is that these positive emotions are mainly linked with the intensity of specular reflections that occur for such kind of glossy materials. This hypothesis is consistent with Fleming (2014) findings who showed that the visual appreciation of a material glossiness is mostly based on visual cues related to characterization of specular reflections. Our hypothesis was confirmed by the objective characterization presented in Section “5 Objective characterization of specular reflections.” Indeed, we showed reported arousal and valence are highly correlated (Pearson linear correlation = 0.939) with the “strength of specular reflections” predictor, computed by state-of-the-art computer vision techniques. This direct link between the intensity of specular reflections and induced emotions is an important result for the perception and design community.

In this context, it is certain that providing the participant the possibility of freely manipulating the mug to examine the visual properties of the object favored the appreciation of the visual qualities produced by the material configuration. This evaluation procedure was considered more natural and made it possible to evaluate more visual characteristics and more particularly specular reflections.

Our results show that, even for an everyday object, usually tagged as emotionally neutral for humans, a simple change on the metalness and smoothness of its material in a virtual environment can trigger a variation on the emotion felt by participants, both in terms of valence and arousal.

These results can also be compared to the values found during the creation of the IAPS standard (International Affective Picture System - Lang et al., 1997). After a scale correction calculation (because we used the same evaluation tool (SAM - Self Assessment Mankini), but not the same unit of measurement), we noticed that the average valence and the arousal scores obtained for the two mug images from the IAPS standard are: Picture mug N° 7009: 0.07_*v*_ and 1.47_*a*_; Picture mug N° 7035: 0.02_v_ and 1.67_a_. These same scores were obtained during a recent evaluation campaign carried out by Bungener et al. (2016), where 150 French participants aged 20–88 evaluated 120 IAPS Pictures. In contrast our mug stimuli lead to valence values in [−0.4, 1.8]_*v*_ and arousal values in [0.6, 2.1]_*a*_. These results suggest that the vision of a mug picture appears neutral in a context of evaluating images selected for their emotional character. But when we look at the valence and arousal scores obtained during our tests which aimed to measure the emotion produced when perceiving the material properties of a 3D object in a virtual environment, we can observe that mugs that have a “smooth, shiny, metallic appearance” gets much higher valence scores. The interval between the lowest valence scores obtained by the “non-metallic and matte” mugs and the highest valence scores obtained by the “metallic and smooth” mugs is almost two units on a 9-point scale. This variation amplitude in measurements found in the evaluations shows that the hardware configurations used to generate these mugs presented sufficient sensory impressions to provoke different emotional responses.

This gap between the evaluation observed in the IAPS standards and in our experience could also be explained by the contribution of the use of virtual reality that allows a participant to hold the mug and to play with the optical properties of the object. The graph presented in Section “4.1 Classification of mugs by valence and arousal” ranks cups by emotional value. In line with the literature, this result once again demonstrates the ability of human beings to visually distinguish materials and to deduce their material properties. In our study, we notice that 6 mugs are considered as unpleasant, one mug as neutral, and 9 mugs as pleasant. Unpleasant mugs obtained very slightly negative average Valence scores (−0.1 to −0.4), and average Arousal scores close to 1 (0.8–0.9). This would indicate that mugs with unpleasant aspects “non-metallic and rough” have been visually perceived as distasteful (arousal) and cause negative feelings (valence). This lack of attractiveness could be explained because these mugs seem bland and therefore gave to the participants the impression of a degraded 3D object. It can also be noticed that the material configuration of these 6 mugs – “non-metallic and rough,” reflect very little light and have a matte appearance that allows very few reflections to pass through. According to our results, it seems that this absence of reflections creates a feeling of dissatisfaction in the observer because this material configuration does not allow the formal aspects to be fully reproduced, which disappear under a slightly blurred appearance. Then, one mug was rated as neutral in appearance with an average Valence score of 0 and the lowest arousal score of the series (0.6). This result would indicate that the material configuration used to generate this mug was neither unpleasant nor pleasant, that is to say emotionally neutral. Finally, we found 9 mugs considered pleasant with average Valence scores of between 0.4 and 1.8 and average Arousal score that can go up to 2.1. This would indicate that it is the “metallic and smooth” material configuration that evokes the most positive emotions. From all these results, our experiment provides new arguments to affirm that humans are capable of discriminating material nuances, and that a minor change in the properties of the material leads to differences in the perceptual impression, as well as stating that changes of these two material properties (*metallicity* and *smoothness*) do provoke different emotional responses. as well as other authors cited above, who like him showed that visual appreciation is based on the estimation of brightness through the properties of reflections, brightness, position and orientation of light sources, appearance of shadows and movement. In this context, it is certain that providing the participant the possibility of freely manipulating the mug to examine the visual properties of the object favored the appreciation of the visual qualities produced by the material configuration. This evaluation procedure was considered more natural and made it possible to evaluate more visual characteristics and more particularly the level of gloss. Our statistical analyzes also revealed that the smoothness property had a slightly stronger impact than the metallic property. This is perhaps explained by the fact that a mug will be in contact with the lips and that the softness attribute of the material is considered important in this context. This observation is very interesting because it confirms that beyond the technical and functional aspects, the choice of material and finish plays a very important role in preferences.

Point that differentiates the two studies lies in the color. Indeed, IAPS images were all in color. Knowing these controversies relating to preference in matters of color, we removed all colored stimulation from our experiment (all our 3D objects were produced in Grayscale No. 128).

Our statistical analyzes also revealed that the smoothness property had a slightly stronger impact than the metallic property. This is perhaps explained by the fact that a mug will be in contact with the lips and that the softness attribute of the material is considered important in this context. This observation is very interesting because it confirms that beyond the technical and functional aspects, the choice of material and finish plays a very important role in preferences.

Our study aimed to enrich knowledge relating to aesthetic emotions. Our results clearly demonstrated the influence of the material properties of a product on hedonic judgment, and the link to perceived specular reflections. The material attribute is highly sought after by consumers because it helps create an emotional identity for products. This notion of attachment to a product had already been demonstrated by Norman (2004, 2013) in his work “Emotional Design, 2004” and “The Design of Everyday Things, 2013.” In this context, the study of material properties turns out to be a determining step in the product design process. Our research provides new elements of answers that will allow designers to better understand the attributes that a product must have to generate pleasure and emotion. These aesthetic pleasures are partly based on visual analysis. To go further, it will be necessary to complete this study by studying all the variables which will allow us to better understand on which material properties this hedonic feeling is based. Is it the amount of light reflected by the object that provokes the best emotional reactions? Is part of this emotion due to reflections from the environment? Is this emotional sensation due to the sharpness of the contours? Among these three factors: color, hue, brightness, and saturation, what is the factor that generates the most emotion? In the race to market ever more attractive products, answers to these questions could be decisive in ensuring the acceptance of a manufactured product.

## 7 Limitation

Given that each stimulus varies along its material dimensions, it is possible that participants produced their rating based on the variation they saw, rather than the strict emotion they felt. A possible way to mitigate this bias would be to introduce additional stimuli with varying appearance properties beyond the studied factor of interest (e.g., color variations).

In this experiment, we measure emotions only through self-reported valence and arousal. Those subjective data could be advantageously completed by objective measures like skin conductance and/or pupil responses. However, its worth noticing that the accurate capture of physiological signals remains an open issue in 6-degrees-of-freedom (6DoFs) VR environments because sensors (e.g., for skin conductance) are very sensitive to user movements. Also, the variation of pupil size is obviously very sensitive to brutal changes in illumination that may occur during the immersive environment exploration.

As “smoothness” and “metalness” factors, we considered built-in parameters from the Unity engine BRDF model; however, these factors do not have a unique definition and may refer to different combinations of physical properties, depending on the underlying BRDF model. For instance, a perceived metallic effect may depend on the intensity of the highlights, the incidence of the Fresnel effect, and the color of the specular reflections. An interesting avenue of improvement would be to consider “perceived” smoothness and metalness factor. For instance, Toscani et al. (2020) propose a perceptual metalness dimension, which can be computed using specific parameters of the ABC BRDF model.

## 8 Conclusion

Since the selection of a material is an important step in the design of an industrial product and that a large part of the interest in a material comes from its visual appearance, the objective of our research was to understand how the visual appearance of a manufactured object influence emotions. We specifically targeted two material characteristics – the smoothness and the metalness, created 16 versions of a mug by modulating these characteristics, and conducted a study in virtual reality. The emotional response, in terms of valence and arousal, produced by these visual stimuli was collected from a panel of 28 participants. Results reveal that both metalness and smoothness have a positive effect on the two measured dimensions of emotional responses, i.e., the mugs with most smooth and metallic surface appearance triggered the strongest positive emotional responses, and conversely for the mugs with opposite characteristics. Using image processing features, we showed that this positive effect is linked to the increasing strength (i.e., sharpness and contrast) of the specular reflections induced by these material properties. Our results confirm that the choice of material for the design of an object can be decisive for a consumer in terms of acceptability, and at least in the case of a mug, designers should opt for a smooth and shiny material to generate more positive emotions.

This experiment could be extended by adding some standard objective measurements such as the measurements of the ocular path and the measurements of the fixation time. This could indeed help to understand the reasons that cause a participant to have different emotions depending on the manipulated material characteristics. Moreover, it could be interesting to intend to measure and characterize the hedonic feelings of participants *during* the manipulation of the 3D objects in the virtual environment, and to evaluate the effects of material variations on this instantaneous measure. Finally, within the framework of the design of an object through both virtual and physical models, it would be interesting to study the emotional response produced in virtual reality with visio-tactile stimulations as physical feedback for the participants.

## Data availability statement

The raw data supporting the conclusions of this article will be made available by the authors, without undue reservation.

## Ethics statement

Ethical approval was not required for the studies involving humans. This study was conducted according to the guidelines of the Declaration of Helsinki and these studies were conducted in accordance with the local legislation and institutional requirements. The data has been anonymized in accordance with the recommendations made by the CNIL. The participants provided their written informed consent to participate in this study.

## Author contributions

CB: Writing – original draft, Conceptualization, Data curation, Formal analysis, Methodology, Supervision, Validation, Writing – review and editing. EZ: Conceptualization, Data curation, Formal analysis, Methodology, Software, Writing – original draft. MG: Methodology, Software, Writing – original draft. JD: Formal analysis, Methodology, Validation, Writing – original draft. PR: Data curation, Formal analysis, Methodology, Validation, Writing – review and editing. GL: Conceptualization, Formal analysis, Funding acquisition, Methodology, Resources, Supervision, Validation, Writing – original draft, Writing – review and editing.
